# LINC01116 Promotes Doxorubicin Resistance in Osteosarcoma by Epigenetically Silencing miR-424-5p and Inducing Epithelial-Mesenchymal Transition

**DOI:** 10.3389/fphar.2021.632206

**Published:** 2021-03-08

**Authors:** Ran Li, Qing Ruan, Jia Zheng, Butian Zhang, Hongliang Yang

**Affiliations:** ^1^ Department of Orthopedics, China-Japan Union Hospital of Jilin University, Changchun, China; ^2^ Department of Radiology, China-Japan Union Hospital of Jilin University, Changchun, China; ^3^ Department of Cardiology, China-Japan Union Hospital of Jilin University, Changchun, China

**Keywords:** LINC01116, miR-424-5p, HMGA2, drug resistance, osteosarcoma

## Abstract

**Background:** Development of resistance to doxorubicin-based chemotherapy limits its curative effect in osteosarcoma. In the current study, we focused on investigating the mechanisms underlying the development of doxorubicin resistance in osteosarcoma.

**Methods:** The human osteosarcoma cell line MG-63 and doxorubicin-resistant MG-63/Dox cells were used in this study. Quantitative real-time polymerase chain reaction (qRT-PCR) was used to detect the expression of the long non-coding RNA LINC01116 in the two cell lines. Then, the specific shRNA for LINC01116 was employed to suppress LINC01116 expression in MG-63/Dox cells. Cell viability was assessed by the CCK-8 and colony formation assays. Cell migration and invasion were evaluated by the transwell assay. Moreover, the epithelial-mesenchymal transition (EMT)-related proteins, E-cadherin, vimentin, and N-cadherin were evaluated by Western blotting. The regulation of LINC01116 on miR-424-5p expression was examined using methylation-specific PCR, RNA immunoprecipitation, and Western blotting assay. The potential targeting of HMGA2 by miR-424-5p was predicted using the bioinformatics databases TargetScan and miRanda and verified by a dual-luciferase reporter assay.

**Results:** LINC01116 was more highly expressed in MG-63/Dox cells than in MG-63 cells. Inhibition of LINC01116 suppressed cell viability, migration, and invasion, along with upregulating the expression of E-cadherin, downregulating vimentin, and attenuating doxorubicin resistance in MG-63/Dox cells. Further mechanism-related investigations indicated that LINC01116 regulated HMGA2 expression via the EZH2-associated silencing of miR-424-5p.

**Conclusion:** LINC01116 exerts regulatory effects on doxorubicin resistance through the miR-424-5p axis, providing a potential approach to overcoming chemoresistance in osteosarcoma.

## Introduction

Osteosarcoma is a type of cancer that originates from the bone and mainly affects the youth. It is the second most fatal cancer in children and young adults, which is often associated with poor prognosis due to cancer progression and metastasis (Ta et al., 2009). The treatment of osteosarcoma is complicated and requires multidisciplinary cooperation, involving complete surgical elimination of all detectable malignant regions as well as multiagent chemotherapy (Ritter and Bielack, 2010). Doxorubicin is one of the most commonly used chemotherapy drugs in osteosarcoma patients (Yang and Zhang, 2013; Hattinger et al., 2017). However, several studies have reported doxorubicin resistance in osteosarcoma (Liu et al., 2016a; Buondonno et al., 2019). As metastasis and drug resistance are both significant challenges in the treatment of osteosarcoma, our study investigated the factors that induce metastasis and doxorubicin resistance in osteosarcoma.

Epithelial-mesenchymal transition (EMT) is a biological process in which non-motile epithelial cells without migration capability transform into a motile phenotype of mesenchymal cells (Pastushenko and Blanpain, 2019). Researchers have shown that EMT is not only related to tumor metastasis and relapse but also affects the drug resistance of malignant cells (Mitra et al., 2015). For instance, the EMT process was found to promote doxorubicin resistance in gastric and breast cancers (Kang et al., 2017; Du et al., 2019). Moreover, it was reported that EMT induced by genetic factors further promoted doxorubicin resistance in hepatocellular carcinoma cells (Xu et al., 2016). Although doxorubicin resistance in osteosarcoma has been studied for years, the underlying genetic mechanisms are complicated and remain unclear.

Through deep RNA sequencing techniques, it has been revealed that there are a series of dynamically regulated microRNAs, non-coding RNAs, and mRNAs that influence osteosarcoma progression and metastasis (Xie et al., 2018). Long non-coding RNAs (lncRNAs), a recently discovered group of non-coding RNAs, play vital roles in the regulation of various biological activities and disease processes (Peng et al., 2017). LncRNAs have also been regarded as essential factors in the development of resistance to anti-cancer chemotherapy. Dysregulation of multiple lncRNA-mediated targets and pathways is thought to contribute to the occurrence of chemoresistance (Chen et al., 2017a). LncRNA LINC01116, also known as TALNEC2, is located in the 2q31.1 region and is involved in the occurrence and development of various tumors (Xu et al., 2017; Hu et al., 2018; Ye et al., 2020). In osteosarcoma, our previous study found that LINC01116 promotes cancer development by regulating the expression of miR-520a-3p and IL6R (Zhang et al., 2018). Zhang et al. also proposed that LINC01116 promotes the development of osteosarcoma by binding to EZH2 to regulate the expression of PTEN and p53 (Zhang et al., 2019b). Furthermore, a recent study showed that LINC01116 contributes to gefitinib resistance in non-small cell lung cancer (Wang et al., 2020a). In lung adenocarcinoma, LINC01116 was found to promote cisplatin resistance via the EMT process (Wang et al., 2020b). However, the exact functions of LINC01116 related to drug response in osteosarcoma remain indistinct.

HMGA2, a gene encoding a protein that belongs to the non-histone chromosomal high mobility group (HMG) protein family, has been reported to promote cancer progression or enhance chemoresistance in various cancers, including lung cancer and gastric cancer (Kumar et al., 2015; Zhu et al., 2017a). HMGA2 overexpression is regarded as a sign of poor prognosis in cancers (Jun et al., 2015). In osteosarcoma, HMGA2 has been reported as a mediator and miRNA target that can affect malignant cell abilities (Liu et al., 2015; He et al., 2016). However, research about its impact on drug resistance is rare. Bioinformatics analysis has revealed that HMGA2 is closely related to LINC01116; thus, we aimed to determine its expression, function, and correlation with LINC01116 to explore whether and how it can influence chemoresistance in osteosarcoma.

In this study, the human osteosarcoma cell line MG-63 and its doxorubicin-resistant strain MG-63/Dox were utilized in several experiments to explore the underlying mechanism of doxorubicin-resistance in osteosarcoma. In addition, the cell invasion, migration, and colony formation capabilities of osteosarcoma cells under different treatments were analyzed to investigate the effects of transcriptional aspects on cancer metastasis. As EMT-associated cancer spread and drug resistance have become an obstacle for osteosarcoma treatments, the regulatory axis of this process, which this research aimed to identify, could be of considerable value for the development of novel therapeutic strategies for metastatic and doxorubicin-resistant osteosarcoma.

## Materials and Methods

### Bioinformatic Analysis

The microarray dataset GSE3362 (including the wild type 143B osteosarcoma cell line and 143B doxorubicin drug resistant cell line) provided by Rajkumar and Yamuna (Rajkumar and Yamuna, 2008) and its related annotation file were downloaded from the Gene Expression Omnibus database (https://www.ncbi.nlm.nih.gov/geo). Bioinformatic analysis was performed using R software (Ver. 3.5.0, https://www.r-project.org/). In brief, the “affy” package (Gautier et al., 2004) was employed for the process of background expression value correction and data normalization. Subsequently, the empirical Bayes moderated *t*-statistics with the “limma” package (Ritchie et al., 2015) were used to screen out the differentially expressed genes (DEGs). The threshold was *p* value <0.05 and log_2_ (fold change) > 1. After the probe annotation, the probes without matching gene symbols were filtered out. At last, "pheatmap" package [https://cran.rstudio.com/web/packages/pheatmap/index.html] was applied to construct a heatmap for the DEGs.

To predict the directly interacting miRNAs of LINC01116, we used the bioinformatics databases miRanda (Enright et al., 2003) and TargetScan (Agarwal et al., 2015). The intersection of the two algorithms was considered as the predicted target miRNAs. Filtering restrictions included a total score ≥140, total energy < 1 kcal/mol, and number of estimated binding sites >1. The candidate mRNAs that may bind to miR-424-5p were also predicted by miRanda and TargetScan.

### Cell Culture and Transfection

The human osteosarcoma cell line MG-63 was purchased from iCell Bioscience Inc. (Shanghai, China). The MG-63 cells were cultured in Minimum Essential Medium (MEM) supplemented with 10% fetal bovine (FBS, Biological Industries) and maintained in a 5% CO_2_ humidified incubator at 37°C.

The doxorubicin-resistant cell line MG-63/Dox was established according to a previous study (Xia et al., 2015). In brief, the MG-63 cell line was gradually exposed to doxorubicin in a stepwise manner (from 5 nM to 100 nM) over a period of 8 months. The cells were incubated in drug-free medium for 1 week before use.

To explore the effects of LINC01116, miR-424-5p, and HMGA2 on doxorubicin resistance in MG-63/Dox cells, sh-LINC01116, miR-424-5p inhibitor, si-EZH2, and HMGA2 overexpression plasmid, respectively, were synthesized and transfected into MG-63/Dox cells using Lipofectamine 2000 reagent (Invitrogen, Carlsbad, CA, United States). The sequences are provided in Supplementary Table S1.

### Cell Counting Kit-8 Assay

MG-63 and MG-63/Dox cells were seeded into 96 well tissue culture plates with 3,000 viable cells per well. To determine the drug resistance of each cell line, cell proliferation was recorded using a concentration gradient of doxorubicin. The differentially treated cells were subjected to the CCK-8 assay after 24 h incubation by adding 10 μL of CCK-8 solution (Dojindo, Kumamoto, Japan) to each well, followed by further incubation for 4 h at 37°C. The absorbance at 450 nm was measured with a microplate photometer.

### Transwell Assay

The migration and invasion of MG-63 and MG-63/Dox cells were evaluated by the transwell assay using 24-well transwell chambers (8 μm, Corning, NY, United States). Cells were maintained in serum-free medium to form a suspension. The upper chambers were supplemented with a 150 μL cell suspension of each cell line and covered with or without Matrigel (Invitrogen, Carlsbad, CA, United States). Then, 500 μL of medium with 10% FBS was added in the bottom chamber. After 24 h of maintenance and washing twice with phosphate buffered saline (PBS), the cells on the upper surface of the membrane were removed by cotton swabs. The migrated or invaded cells were fixed with 95% ethanol, stained with crystal violet for 10 min, counted in randomly selected fields, and photographed under a microscope.

### Western Blot

Total protein of MG-63 and MG-63/Dox cells was extracted with the RIPA lysis buffer (Beyotime, Shanghai, China) and quantified using an enhanced BCA Protein Assay Kit (Beyotime, Shanghai, China). Then, 20 μg of total protein was separated by SDS-PAGE and transferred to PVDF membranes. Next, 5% bovine serum albumin (BSA, Sigma-Aldrich) was added for 30 min under room temperature to block non-specific antigens. Subsequently, the membranes were incubated with primary antibodies overnight at 4°C (GAPDH was used as an internal reference), followed by the addition of the corresponding secondary antibody. After 1 h of incubation at room temperature, HRP-labelled proteins were detected using a BeyoECL Star Kit (Beyotime) and filmed after washing with TBST three times. The primary antibodies used were rabbit anti-E-cadherin (ab40772, 1:10,000), rabbit anti-vimentin (ab92547, 1:1,000), rabbit anti-N-cadherin (ab18203, 1 μg/ml), rabbit anti-HMGA2 (ab97276, 1:1,000), and rabbit, rabbit anti-EZH2 (ab191250, 1:1,000) and anti-GAPDH (ab8245, 1:4,000). The secondary antibody was goat anti-rabbit IgG H&L (HRP) (ab205718, 1:2,000). All antibodies were purchased from Abcam (MA, United States). Proteins were visualized by ECL-plus reagents (Millipore, Billerica, MA, United States), and the band density was measured using Image J software (Version1.48u, Bethesda, United States).

### Quantitative Real-Time PCR and Methylation-specific PCR

After isolating total RNA with TRIzol reagent (Invitrogen), the RNA quality and concentration were confirmed by NanoDrop 2000 (Thermo Fisher Scientific Inc., United States). Then, a SuperScript^TM^ III Reverse Transcriptase Kit (Invitrogen, Carlsbad, CA, United States) was employed for lncRNA/mRNA reverse transcription, whereas the miRCURY LNA RT Kit (Qiagen, Duesseldorf, Germany) was utilized for miRNA reverse transcription. qRT-PCR was conducted using THUNDERBIRD SYBR^®^ qPCR Mix (Toyobo, Japan) on the LightCycler 480 PCR System (Roche, Rotkreuz, Switzerland). LncRNA and mRNA expression levels were normalized against GAPDH while miRNA expression values were normalized against U6. Relative RNA expression was calculated using the 2^−ΔΔCt^ method. The primer sequences used for qRT-PCR are listed in Supplementary Table S2.

The methylation status of the miR-424-5p promoter CpG island was detected using MSP. The genomic DNA was isolated from cells using the QIAamp DNA Mini Kit (Qiagen) and was treated with bisulfite using the Epitect Bisulfite Kit (Qiagen), according to the manufacturer’s instructions. Then, the PCR reaction was performed on the bisulfite-treated DNA. Methylation or unmethylation primers for MSP are listed in Supplementary Table S3.

### Colony Formation Assay

Differentially transfected MG-63/Dox cells were seeded in six-well plates containing MEM medium with 10% FBS and incubated in a humidified atmosphere containing 5% CO_2_ at 37°C. After 2 weeks, the cells were washed with PBS and fixed with 4% paraformaldehyde for 15 min. Then, the cells were washed again and incubated with 0.1% aqueous crystal violet solution for 15 min, followed by washing with sterile deionized water. Individual colonies were counted and photographed under a microscope.

### RNA Immunoprecipitation Assay

The RIP assay was performed using the Magna RIP RNA-Binding Protein Immunoprecipitation Kit (Millipore) according to the manufacturer’s instructions. In brief, MG-63/Dox cells were first lysed and then incubated with anti-EZH2 (ab191250, Abcam) or rabbit IgG that conjugated to magnetic beads at 4°C overnight. After washing of the unbound materials, immunoprecipitated RNA molecules were purified and were detected using qRT-PCR.

### Dual-Luciferase Reporter Assay

The specific sequences of LINC01116 and HMGA2 3-untranslated region (3-UTR) harboring the complementary site of miR-424-5p were cloned into the pmirGLO vector (Promega, Madison, WI, United States) to generate wild-type (WT) and mutant (MUT) LINC01116 and HMGA2 firefly luciferase plasmids, respectively. The Renilla luciferase reporter vector was used as an internal control. PmirGLO vector, LINC01116-WT, LINC01116-MUT, HMGA2-WT, and HMGA2-MUT were co-transfected with miR-424-5p mimics or miR-424-5p mimics NC into 293T cells using the Lipofectamine 2000 reagent. After 48 h, the reporter activities were measured using the Dual Luciferase Assay System (Promega). The ratio of firefly luciferase signal to Renilla luciferase signal was calculated for the relative luciferase activity.

### Statistical Analysis

All experiments in this study were independently repeated three times. Data are shown as mean values ± standard deviations (SD) and were analyzed using GraphPad Prism version 6.0 (GraphPad Software, CA, United States). Differences between two groups were evaluated by the Student’ s two-tailed unpaired *t*-test, while the differences between three or more groups were evaluated by the one-way ANOVA. *p* value less than 0.05 was considered as statistically significant.

## Results

### LINC01116 is up-Regulated and Influences Chemo-Resistance in MG-63/Dox Cells

**FIGURE 1:**
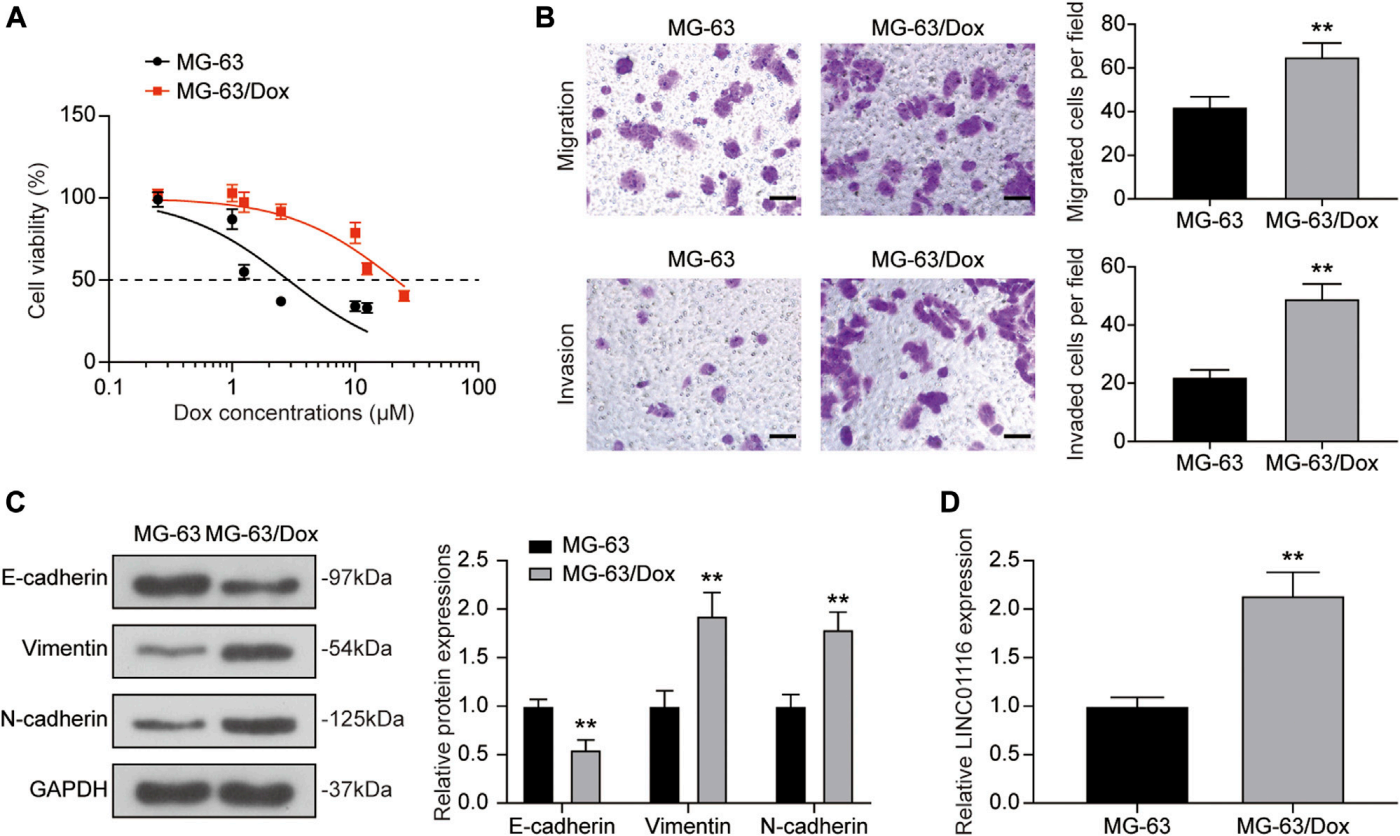
LINC01116 expression was up-regulated in the doxorubicin resistant MG-63/Dox cells compared to the parental MG-63 cells. **(A)** Cells were exposed to a concentration gradient of doxorubicin (0.25, 1, 1.25, 2.5, 10, 12.5 μM for MG-63 cells, 0.25, 1, 1.25, 2.5, 10, 12.5, 25 μM for MG-63/Dox cells) for 48 h. Then the cell viability and 50% inhibitory concentration (IC_50_) of doxorubicin were evaluated using the CCK-8 assay. **(B)** Transwell assay for migration and invasion abilities of MG-63 and MG-63/Dox cells. Scale bar: 50 μm. **(C)** Western blot for E-cadherin, vimentin, and N-cadherin protein expression in MG-63 and MG-63/Dox cells. **(D)** Quantitative real-time PCR detection of LINC01116 expression in MG-63 and MG-63/Dox cells. Data are expressed as mean ± SD (n = 3). **p < 0.01, compared with the MG-63 cells.

The CCK-8 assay was used to confirm the doxorubicin resistance of MG-63/Dox cells in comparison to the parental MG-63 cells. As shown in Figure 1A, the IC_50_ value for MG-63/Dox cells (21.54 μM) was significantly higher than that for parental MG-63 cells (2.87 μM). Furthermore, according to the transwell assay results, cell migration and invasion were both increased in MG-63/Dox cells compared to MG-63 cells (Figure 1B). Western blotting results indicated that E-cadherin expression was suppressed while vimentin and N-cadherin levels were upregulated in MG-63/Dox cells compared to parental MG-63 cells (Figure 1C), suggesting that doxorubicin-resistance is related to EMT promotion in osteosarcoma. In addition, we evaluated LINC01116 expression in MG-63 and MG-63/Dox cells. There are 4 isoforms for LINC01116 (LINC01116-201, -202, -203, -204), and we selected the longest isoform, LINC01116-202. The results showed that the relative expression of LINC0116 was remarkably increased in MG-63/Dox cells compared to the parental MG-63 cells (Figure 1D).

### Knockdown of LINC01116 Suppressed the Proliferation, Migration, and Invasion of MG-63/Dox Cells

**FIGURE 2:**
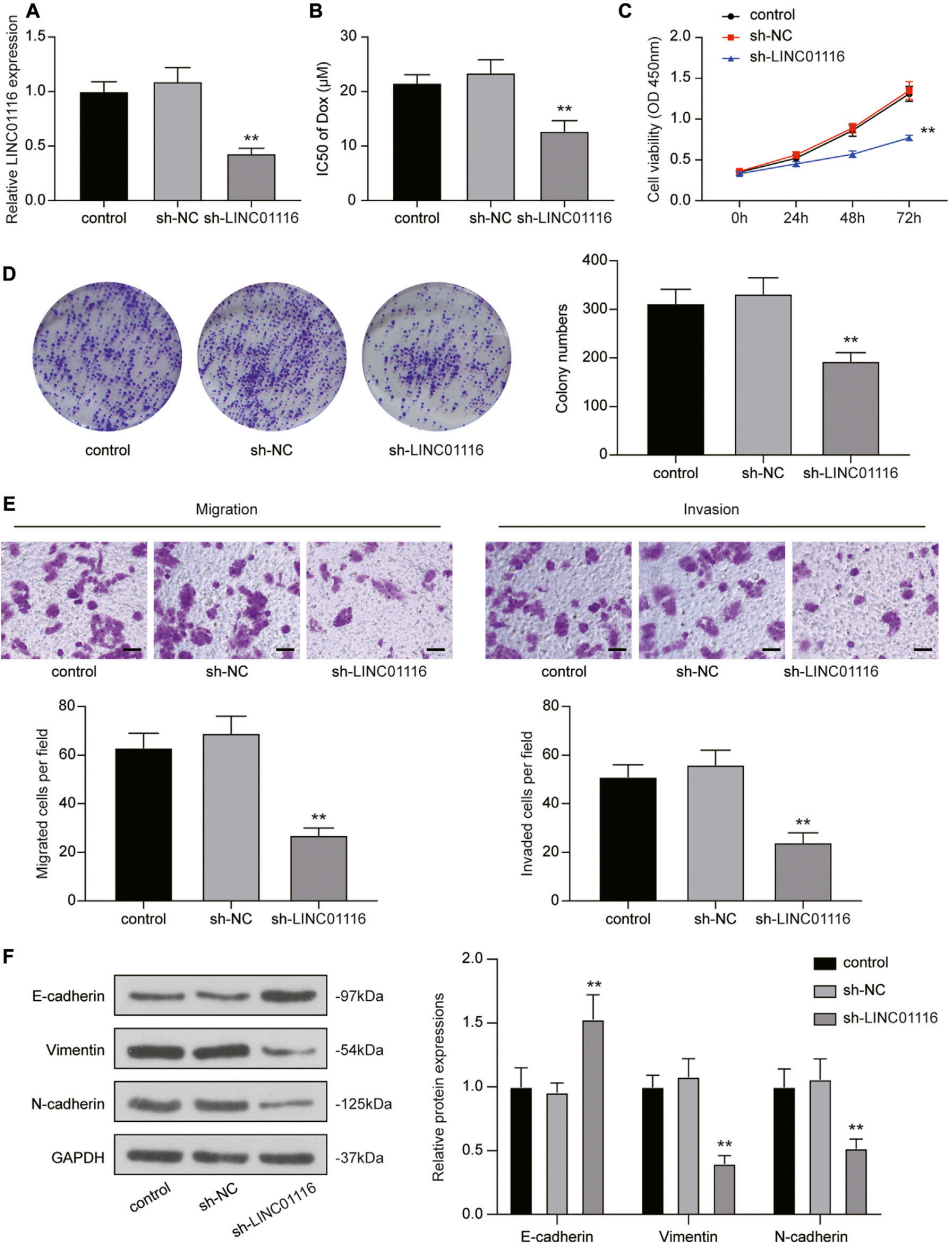
Knockdown of LINC01116 inhibited the proliferation, migration, and invasion of MG-63/Dox cells. **(A)** Quantitative real-time PCR detection of LINC01116 expression in MG-63/Dox cells transfected with sh-LINC01116 or sh-NC. **(B)** sh-LINC01116 transfected MG-63/Dox cells were treated with a concentration gradient of doxorubicin for 48 h, and IC_50_ of doxorubicin was calculated using the CCK-8 assay. **(C)** CCK-8 assay for cell viability of the transfected MG-63/Dox cells. **(D)** Colony formation assay for assessing cell proliferation of the transfected MG-63/Dox cells. **(E)** Transwell assay for assessing migration and invasion of the transfected MG-63/Dox cells. Scale bar: 50 μm. **(F)** Western blot for detection of E-cadherin, vimentin, and N-cadherin protein expression in MG-63/Dox cells transfected with sh-NC or sh-LINC01116 or without transfection. Data are expressed as mean ± SD (n = 3). **p < 0.01, compared with the sh-NC group.

To investigate the effects of LINC01116 on MG-63/Dox cells, LINC01116 shRNA was transfected into the MG-63/Dox cells to downregulate LINC01116 expression. qRT-PCR results verified that LINC01116 expression was suppressed in the sh-LINC01116 group (Figure 2A). Additionally, the IC_50_ value of doxorubicin in the MG-63/Dox cells demonstrated a decrease and was remarkably lower than in the control group and sh-NC group (Figure 2B), suggesting that LINC01116 plays a vital role in the drug resistance of MG-63/Dox cells. Furthermore, the CCK-8 assay and colony formation assay results indicated that cell proliferation was inhibited in MG-63/Dox cells transfected with sh-LINC01116 (Figures 2C,D). Additionally, the migration and invasion capabilities of MG-63/Dox cells were decreased by LINC01116 downregulation (Figure 2E). Western blot results revealed that the knockdown of LINC01116 resulted in increased E-cadherin expression and reduced vimentin and N-cadherin expression in MG-63/Dox cells (Figure 2F). Taken together, LINC01116 knockdown suppressed the proliferation, inhibited the migration and invasion, and reversed the EMT process in MG-63/Dox cells.

### LINC01116 Directly Targets miR-424-5p and Subsequently Regulates HMGA2 Expression

**FIGURE 3:**
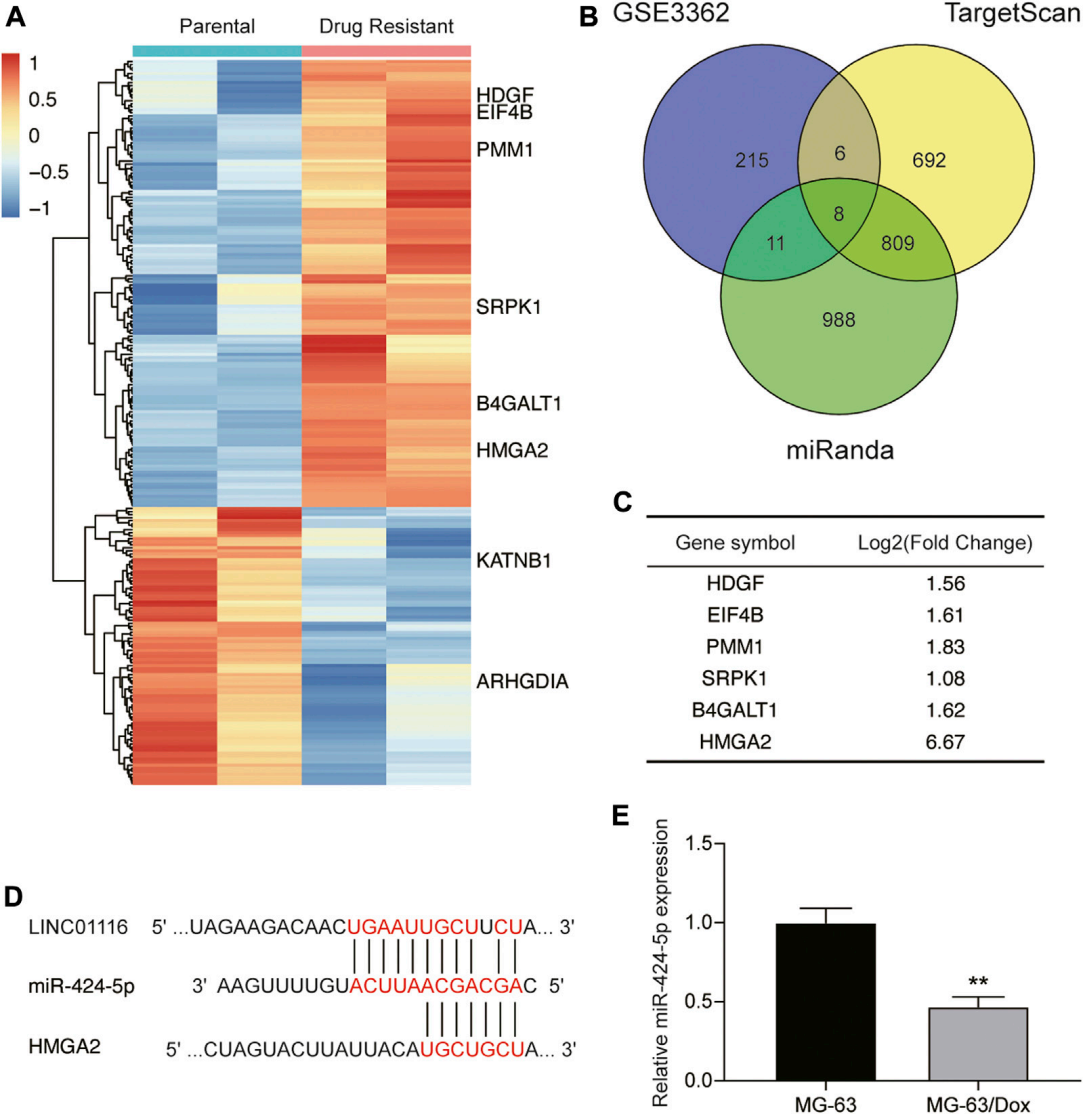
LINC01116 directly targets miR-424-5p and subsequently regulates HMGA2 expression. **(A)** Heatmap of the 240 significant differentially expressed genes in the microarray dataset GSE3362. Horizontal axis shows the samples, while the vertical axis denotes the differentially expressed genes. The histogram represents the color gradient, and each rectangle represents the expression value of each sample. **(B)** Venn diagram showing the eight overlapping mRNAs which were differentially expressed in the microarray dataset GSE3362, and were predicted to be the targets of miR-424-5p by the miRanda and TargetScan algorithms. **(C)** Expression changes in the six significantly upregulated genes (HDGF, EIF4B, PMM1, SRPK1, B4GALT1, and HMGA2) in the doxorubicin resistant cell line. **(D)** The binding sites between LINC01116, miR-424-5p, and HMGA2. **(E)** Quantitative real-time PCR detection of miR-424-5p expression in MG-63 and MG-63/Dox cells. Data are expressed as mean ± SD (n = 3). **p < 0.01, compared with the MG-63 cells.

Next, we applied the miRanda and TargetScan algorithms to predict the downstream target of LINC01116, and selected miR-424-5p for further research. We also performed bioinformatics analysis to identify the differentially expressed genes in the microarray dataset GSE3362, which included the wild type 143B osteosarcoma cell line (n = 2) and the 143B doxorubicin drug resistant cell line (n = 2). As demonstrated in Figure 3A, we identified 240 genes that were differentially expressed between the parental cell line and doxorubicin drug-resistant osteosarcoma cell line. We further shortlisted a total of eight mRNAs (upregulated: HDGF, EIF4B, PMM1, SRPK1, B4GALT1, and HMGA2; downregulated: KATNB, ARHGDIA) by overlapping the differentially expressed genes with the mRNAs predicted to interact with miR-424-5p by the miRanda and TargetScan algorithms (Figure 3B). The changes in the expression levels of the upregulated genes are shown in Figure 3C. We subsequently evaluated the expression of HDGF, EIF4B, PMM1, SRPK1, B4GALT1, and HMGA2 in MG-63 and MG-63/Dox cells by qRT-PCR. Among them, we selected HMGA2 as it showed the highest fold-upregulation in MG-63/Dox cells (Supplementary Figure S1A). Figure 3D demonstrates the target binding sites between LINC01116, miR-424-5p, and HMGA2. In addition, miR-424-5p expression was repressed in MG-63/Dox cells compared to the parental MG-63 cells (Figure 3E).

### LINC01116 Directly Interacted with EZH2 and Suppressed miR-424-5p Expression

**FIGURE 4:**
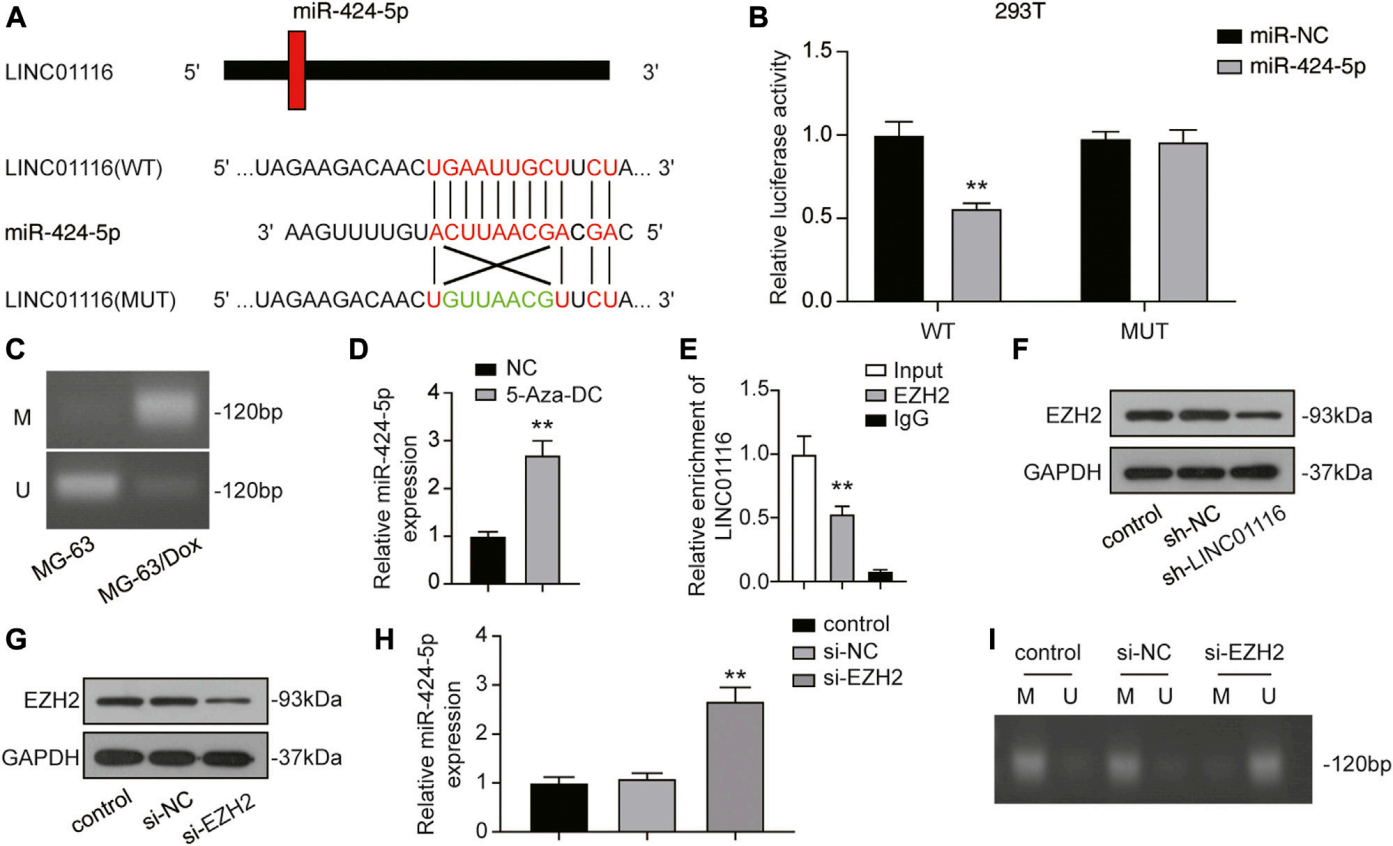
LINC01116 negatively regulated miR-424-5p. **(A)** Construction of wild-type or mutant LINC01116 3′UTR luciferase reporter vectors is demonstrated. The wild type vector for LINC01116 consists of a miR-424-5p binding region, whereas the mutant vector for LINC01116 does not. **(B)** The binding site between LINC01116 and miR-424-5p was verified by a dual-luciferase reporter assay. **(C)** Methylation-specific PCR detection of the CpG methylation within the promoter region of miR-424-5p in MG-63 and MG-63/Dox cells. **(D)** Quantitative real-time PCR detection of miR-424-5p expression in MG-63/Dox cells after 5-Aza-DC treatment. **(E)** RNA immunoprecipitation assay for detection of the interaction between LINC01116 and EZH2 in MG-63/Dox. IgG was used as the negative control. The binding of LINC01116 to EZH2 or to IgG is presented as a ratio of the total LINC01116 from the input. **(F)** Western blot for detection of EZH2 in MG-63/Dox cells transfected with sh-NC or sh-LINC01116 or without transfection. **(G)** Western blot for detection of EZH2 in MG-63/Dox cells transfected with si-NC or si-EZH2 or without transfection. **(H)** Quantitative real-time PCR detection of miR-424-5p expression in MG-63/Dox cells transfected with si-NC or si-EZH2 or without transfection. **(I)** Methylation-specific PCR detection of the CpG methylation within the promoter region of miR-424-5p in MG-63/Dox cells transfected with si-NC or si-EZH2 or without transfection. **(I)** Methylation-specific PCR detection of the CpG methylation within the promoter region of miR-424-5p in MG-63/Dox cells transfected with si-NC or si-EZH2 or without transfection. Data are expressed as means ± SD (n = 3). U: unmethylated, M: methylated. **p < 0.01, compared with the NC group or IgG group.

We performed a dual-luciferase reporter assay to confirm the binding sites of LINC01116 and miR-424-5p. After overexpression of miR-424-5p, the luciferase activity of wild-type LINC01116 was significantly inhibited. However, miR-424-5p overexpression exhibited limited effects on the luciferase activity of mutant LINC01116 (Figures 4A,B). These results indicate that miR-424-5p can directly bind to the LINC01116 binding site. The upregulated LINC01116 inhibited the miR-424-5p expression in MG-63/Dox cells. Moreover, miR-424-5p expression is reportedly affected by promoter methylation (Jin et al., 2017), suggesting that the ceRNAs theory was not the only underlying mechanism of its downregulation in MG-63/Dox cells. Thus, we conducted the methylation-specific PCR in MG-63 and MG-63/Dox cells to evaluate the methylation status of the miR-424-5p promoter region. The results indicated that the miR-424-5p promoter was hypermethylated in MG-63/Dox cells, but was hypomethylated in the MG-63 cells (Figure 4C). The MG-63/Dox cells were then treated with the nonspecific demethylation agent 5-aza-2′-deoxycytidine (5-Aza-DC), and the results indicated that miR-424-5p expression was significantly increased (Figure 4D). These results suggest that decreased miR-424-5p expression is associated with promoter hypermethylation in MG-63/Dox cells. Moreover, LINC01116 reportedly interacts directly with the epigenetic regulator EZH2 (Zhang et al., 2019b); therefore, we tried to figure out if LINC01116 could regulate the methylation pattern via interaction with EZH2 in MG-63/Dox cells. The RIP assay results confirmed that LINC01116 could directly interact with EZH2 in MG-63/Dox cells (Figure 4E). Moreover, the Western blot results revealed that LINC01116 knockdown resulted in reduced EZH2 expression in MG-63/Dox cells (Figure 4F). In addition, we evaluated the expression of miR-424-5p after knocking down EZH2 in MG-63/Dox cells (Figure 4G). The results indicated that miR-424-5p could be induced by inhibiting EZH2 (Figure 4H). Furthermore, EZH2 inhibition resulted in the suppressed hypermethylation of the miR-424-5p promoter. Taken together, these results show that miR-424-5p could be negatively regulated by LINC01116.

### LINC01116 Exerts Effects on MG-63/Dox Cells by Suppressing miR-424-5p Expression

**FIGURE 5:**
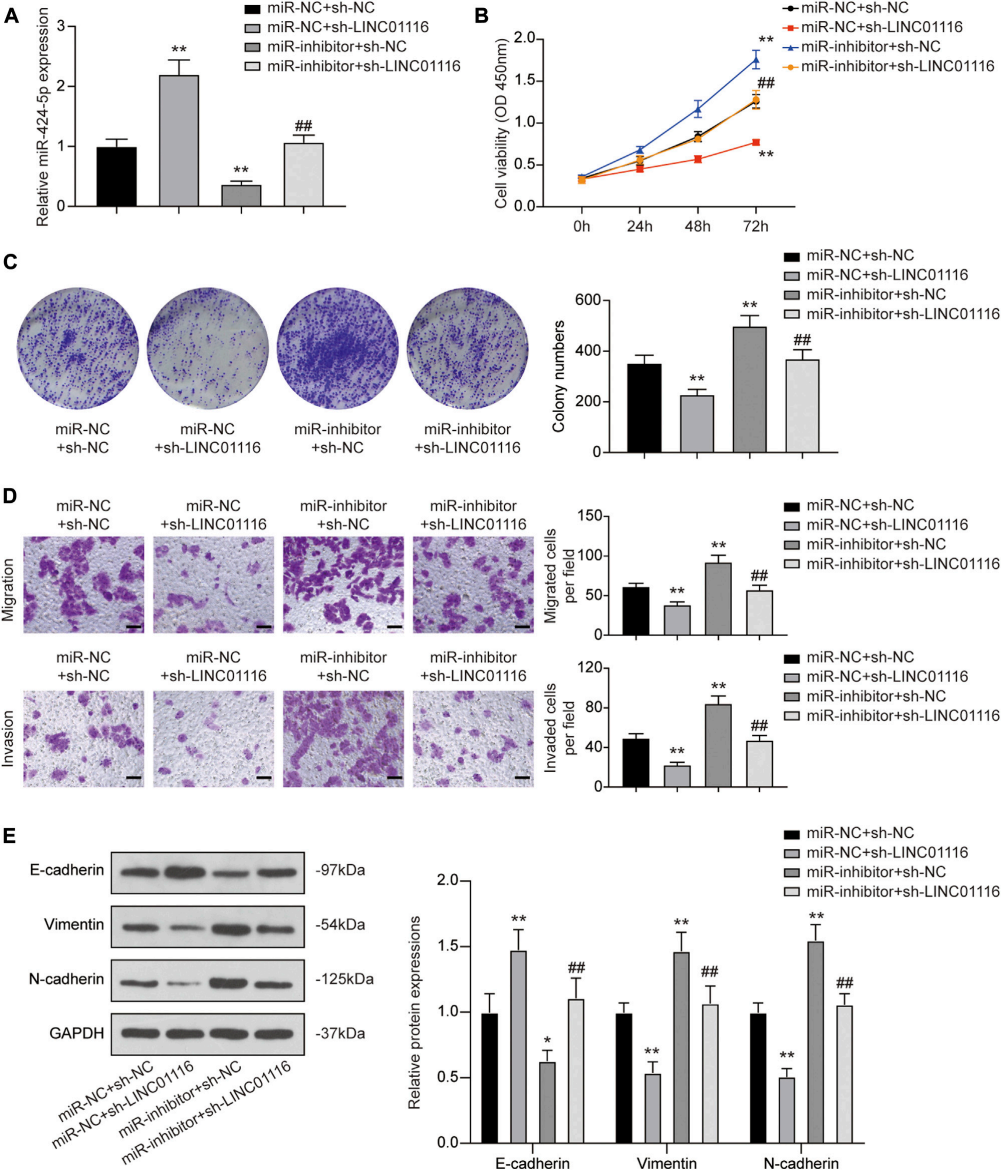
LINC01116 regulated proliferation, migration, invasion, and EMT in MG-63/Dox cells through miR-424-5p. **(A)** Quantitative real-time PCR detection of miR-424-5p expression in MG-63/Dox cells after transfection. **(B)** CCK-8 assay for assessing cell viability of MG-63/Dox cells after transfection. **(C)** Colony formation assay for evaluating cell proliferation of MG-63/Dox cells after transfection. **(D)** Transwell assay for assessing cell migration and invasion of MG-63/Dox cells after transfection. Scale bar: 50 μm. **(E)** Western blot for E-cadherin, vimentin, and N-cadherin protein expression in MG-63/Dox cells after transfection. Data are expressed as means ± SD (n = 3). *p < 0.05, **p < 0.01, compared with the miR-NC group or miR-NC + sh-NC group. ^##^ *p* < 0.01, compared with the miR-inhibitor + sh-NC group.

To confirm the effects of LINC01116 and miR-424-5p on MG-63/Dox cells, we performed the rescue assay. MiR-424-5p expression was promoted when MG-63 cells were transfected with sh-LINC01116. In addition, a specific inhibitor targeting miR-424-5p reversed the sh-LINC01116-induced upregulation of miR-424-5p expression (Figure 5A). Furthermore, miR-424-5p inhibition reversed the effects of sh-LINC01116 on cell proliferation, migration, invasion, and EMT in MG-63/Dox cells (Figures 5B–E). Thus, miR-424-5p could rescue the effect of sh-LINC01116 on cell proliferation in MG-63/Dox cells.

### LINC01116 Regulates the EMT Process Through the LINC01116/miR-424-5p/HMGA2 Axis

**FIGURE 6:**
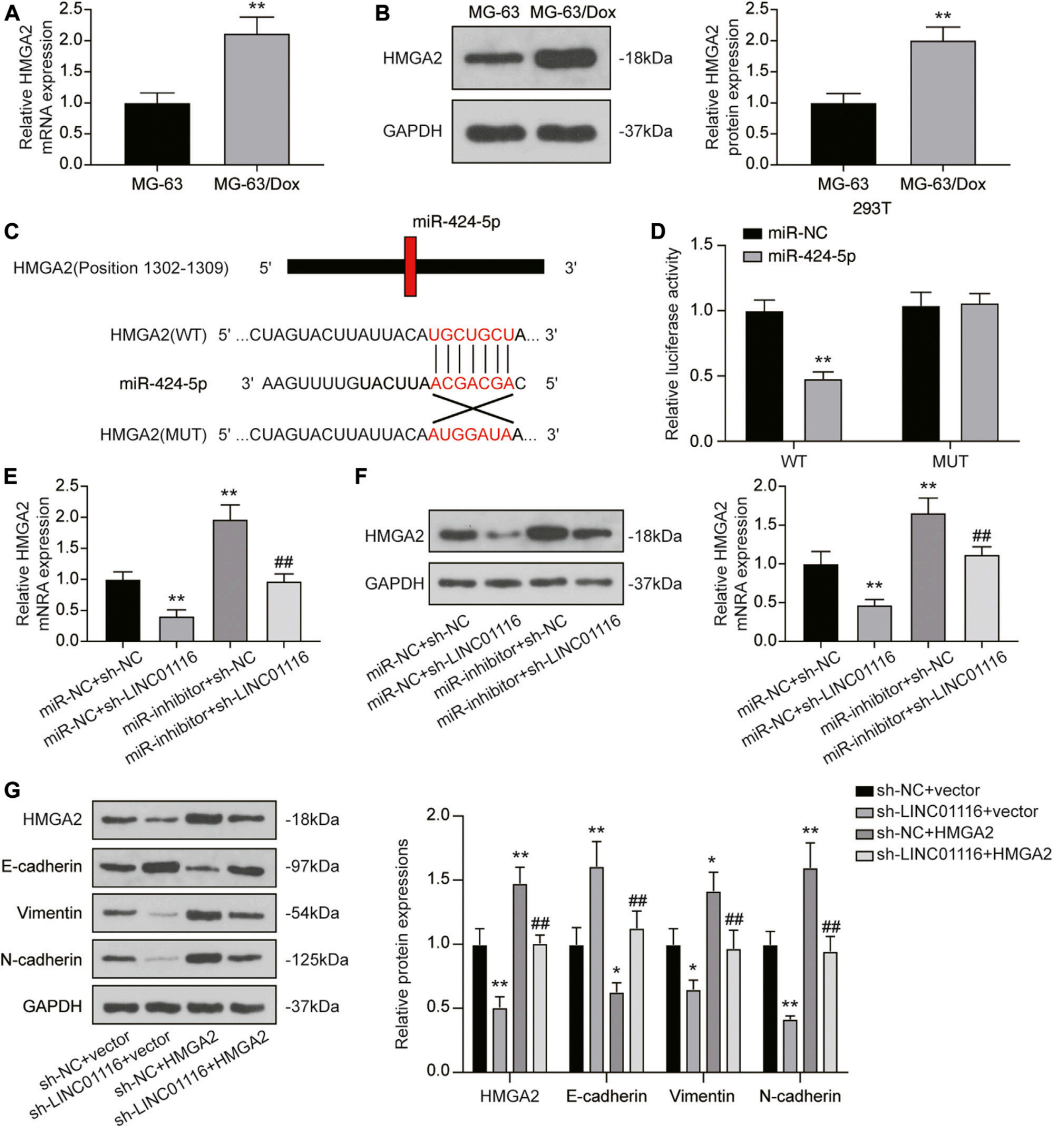
LINC01116/miR-424-5p/HMGA2 axis influences the EMT process in MG-63/Dox cells. **(A)** Quantitative real-time PCR detection of HMGA2 mRNA expression in MG-63 and MG-63/Dox cells. **(B)** Western blot for HMGA2 protein expression in MG-63 and MG-63/Dox cells. **(C)** Construction of wild-type or mutant HMGA2 3′UTR luciferase reporter vectors is demonstrated. The wild type vector for HMGA2 consists of a miR-424-5p binding region, whereas the mutant vector for HMGA2 does not. **(D)** The binding site between miR-424-5p and HMGA2 was verified by a dual-luciferase reporter assay. **(E)** Quantitative real-time PCR detection of HMGA2 mRNA expression in MG-63/Dox cells after transfection. **(F)** Western blot for HMGA2 protein expression in MG-63/Dox cells after transfection. **(G)** Western blot for HMGA2, E-cadherin, vimentin, and N-cadherin protein expression in MG-63/Dox after transfection. Data are expressed as means ± SD (n = 3). *p < 0.05, **p < 0.01, compared with the miR-NC + sh-NC group or sh-NC + vector group. ^##^ *p* < 0.01, compared with the miR-inhibitor + sh-NC group or sh-NC + HMGA2 group.

As HMGA2 was found to be downstream of miR-424-5p, we detected HMGA2 expression in MG-63 and MG-63/Dox cells. The results indicated that both mRNA and protein expression of HMGA2 was upregulated in MG-63/Dox cells compared to MG-63 cells (Figures 6A,B). Through the dual-luciferase reporter assay, we also confirmed that miR-424-5p could target HMGA2 (Figures 6C,D). In addition, qRT-PCR and western blot results revealed that HMGA2 expression was downregulated when MG-63/Dox cells were transfected with sh-LINC01116 and upregulated when the cells were transfected with the miR-424-5p inhibitor. Co-transfection of sh-LINC01116 and miR-424-5p inhibitor in MG-63/Dox cells had little effect on HMGA2 expression (Figures 6E,F). Furthermore, the expression of epithelial-related protein E-cadherin, and fibroblast-related proteins vimentin and N-cadherin was analyzed in MG-63/Dox cells transfected with sh-LINC01116 or the HMGA2 overexpression vector. The results indicated that E-cadherin expression was downregulated while vimentin and N-cadherin were upregulated when HMGA2 was overexpressed. Moreover, HMGA2 overexpression reversed the effects of LINC01116 inhibition on the EMT process in MG-63/Dox cells (Figure 6G). To conclude, LINC01116 is upregulated in the doxorubicin-resistant MG-63/Dox cells. LINC01116 promotes MG-63/Dox cell proliferation, migration, and invasion by binding to miR-424-5p to regulate HMGA2 expression and activate the EMT process. Through the EMT process, the epithelial cells in tumor tissues transform into fibroblasts, leading to cell invasion and doxorubicin resistance in osteosarcoma.

## Discussion

Osteosarcoma is the most common bone-derived tumor in children and adolescents, and patients with advanced osteosarcoma with signs of metastasis have a poor prognosis (Brown et al., 2017). Chemo-resistance and metastasis are significant problems that need to be solved for successful osteosarcoma treatment (Ritter and Bielack, 2010). Therefore, this study focused on investigating their underlying mechanism.

Long non-coding RNAs are now regarded as significant in the progression, metastasis, and prognosis of osteosarcoma (Yang et al., 2016; Chen et al., 2017b). Several lncRNAs have been reported to be differentially expressed in doxorubicin-resistant osteosarcoma cells and tissues (Zhu et al., 2017b; Zhang et al., 2017; Hu et al., 2018). In this study, we established the doxorubicin-resistant MG-63/Dox cell line to investigate the LINC01116-mediated molecular mechanisms of doxorubicin resistance in osteosarcoma. LINC01116 has been reported to be an oncogene in multiple cancers (Xu et al., 2017; Hu et al., 2018; Ye et al., 2020), including osteosarcoma (Zhang et al., 2018; Zhang et al., 2019b). Moreover, recent studies have demonstrated that LINC01116 increases cisplatin resistance in lung adenocarcinoma (Wang et al., 2020b) and gefitinib resistance in non-small cell lung cancer (Wang et al., 2020a). Consistent with previous studies, we found that LINC01116 contributed to doxorubicin resistance in osteosarcoma. LINC01116 expression was higher in MG-63/Dox cells than in parental MG-63 cells. Knockdown of LINC01116 inhibited cell proliferation, migration, invasion, and EMT in MG-63/Dox cells.

In recent years, the hypothesis of ceRNAs related to lncRNAs has received widespread attention in the research of disease pathology. By binding to miRNAs, lncRNAs can modulate the expression and function of miRNA-targeted mRNAs (Yoon et al., 2014). Previous studies have demonstrated that LINC01116 regulates a number of miRNAs and mRNAs in various diseases. For example, Yuan et al*.* reported that LINC01116 regulated keloid formation through the miR-203/SMAD5 axis (Yuan et al., 2020). In colorectal cancer, LINC01116 was found to promote cancer progression through the miR-9-5p/STMN1 axis (Bi et al., 2020). In brain glioma, LINC01116 regulates VEGFA expression through competitive absorption of miR-31-5p (Ye et al., 2020). Similarly, LINC01116 promotes the progression of oral squamous cell carcinoma through miR-136-mediated FN1 overexpression (Chen et al., 2019). In breast cancer, LINC01116 directly combines with miR-145 to increase ESR1 expression, and subsequently promotes tumor development (Hu et al., 2018). Moreover, by regulating the miR-520a-3p/IL6R axis in osteosarcoma, LINC01116 promotes cell viability and migration (Zhang et al., 2018). It is worth noting that miR-520a-3p could reverse gefitinib resistance in non-small cell lung cancer (Liu et al., 2016b). Therefore, based on the above evidence, LINC01116 might play a critical role in doxorubicin resistance in osteosarcoma. However, the detailed mechanism remains unknown.

In this study, using the miRanda and TargetScan algorithms, we confirmed that miR-424-5p is the target of LINC01116. MiR-424-5p has been found to be downregulated in various cancers (Zhang et al., 2014; Wang et al., 2016; Liu et al., 2018; Wu et al., 2019). However, there are few reports about the role of miR-424-5p in osteosarcoma. Vimalraj et al. demonstrated that melatonin upregulated the expression of miR-424-5p to suppress tumor angiogenesis, while modulating surrounding endothelial cell proliferation and migration in osteosarcoma (Vimalraj et al., 2020). Another study found that miR-424-5p was downregulated in doxorubicin-resistant osteosarcoma cells (Gu et al., 2020). In line with the previous study, our study showed that miR-424-5p was suppressed in MG-63/Dox cells compared to parental MG-63 cells. The MiR-424-5p inhibitor abolished the inhibitory effects of LINC01116 on cell proliferation, migration, invasion, and EMT in MG-63/Dox cells. Thus, these results indicate that LINC01116 promotes doxorubicin resistance in osteosarcoma cells by targeting miR-424-5p.

Furthermore, HMGA2 was identified downstream of the LINC01116/miR-424-5p axis in this study. As mentioned before, HMGA2 has been reported to enhance cancer progression and drug resistance in several cancers, which eventually leads to poor prognosis (Zhang et al., 2019a). Similar to its role in other malignancies, we found that the expression of HMGA2 was upregulated in MG-63/Dox cells compared to parental MG-63 cells, and its overexpression could reverse the effects of LINC01116 inhibition on the EMT process. Overall, the results revealed that LINC01116 promoted doxorubicin resistance via the miR-424-5p/HMGA2 axis in MG-63/Dox cells.

EMT, as a vital driver of cancer metastasis, has been studied for decades. Focusing on the regulation of EMT has become a novel approach for anti-tumour therapies (Lo and Zhang, 2018). In this study, an in-depth investigation of EMT in osteosarcoma cells indicated that upregulated LINC01116 induced miR-424-5p suppression and downstream HMGA2 overexpression, which promoted the EMT process and subsequent cancer metastasis. The dismal prognosis and poor survival level of osteosarcoma have been proven to be mainly due to distal metastasis, especially pulmonary metastasis (Han et al., 2019); thus, the findings of the present study could be valuable for the clinical prevention of osteosarcoma metastasis and relapse.

Furthermore, promoter hypermethylation is another important mechanism for the decreased miRNA expression during chemoresistance in osteosarcoma (Jin et al., 2017; Li et al., 2017; Wang et al., 2019; Jin et al., 2020). Therefore, we speculate that promoter hypermethylation is a potential mechanism of miR-424-5p reduction in doxorubicin-resistant osteosarcoma. In this study, we found that promoter hypermethylation contributed to the decreased expression of miR-424-5p in MG-63/Dox cells. A previous study also reported that LINC01116 could directly integrate with EZH2 to regulate the demethylation modification of H3K4me2 in p53 and PTEN (Zhang et al., 2019b). Here, we confirmed that LINC01116 could directly interact with EZH2. The inhibition of LINC01116 suppressed EZH2 expression, alleviating the promoter hypermethylation of miR-424-5p and inducing its expression. This epigenetic regulatory pathway helps to propose additional mechanisms for LINC01116 and its regulation of miR-424-5p expression.

To conclude, our study explains the mechanisms by which the LINC01116/miR-424-5p/HMGA2 axis influences the EMT process and subsequent metastasis as well as doxorubicin resistance in MG-63/Dox cells. Modulation of this axis could be utilized for novel adjuvant treatment for anti-osteosarcoma chemotherapy.

### Conclusion

In summary, LINC01116 was upregulated in the doxorubicin-resistant osteosarcoma cell line MG-63/Dox. The inhibition of LINC01116 suppressed cell proliferation, migration, invasion, and EMT in MG-63/Dox cells by regulating to miR-424-5p to inhibit HMGA2 expression. Our findings support the oncogenic role of LINC01116 in promoting the development of doxorubicin resistance in osteosarcoma, and LINC01116/miR-424-5p/HMGA2 axis may be a promising chemo sensitizing strategy for the treatment of osteosarcoma.

### Limitations and Future Direction of the Study

First of all, LINC01116 has four isoforms, and this study only investigated the longest one. The other three isoforms should also be considered in further studies. Also, the effects of LINC01116 were only confirmed in a single cell line in the present study. To make the conclusion more reliable, further studies need to be performed on additional osteosarcoma cell lines. In addition, the *in vitro* cell experiments limited the strength of our work. Further studies should include a xenograft model to confirm the effect of LINC01116 inhibition on the *in vivo* sensitivity to doxorubicin. Moreover, the mechanism by which LINC01116 influences doxorubicin resistance in osteosarcoma is complex, and the precise mechanism of LINC01116 expression will be the focus of our future research.

## Data Availability

The original contributions presented in the study are included in the article/Supplementary Material, further inquiries can be directed to the corresponding author/s.

## Abbreviations:

EMT, epithelial-mesenchymal transition; qRT-PCR, Quantitative real-time polymerase chain reaction; lncRNA, long no-coding RNAs; HMG, high mobility group; DEGs, differentially expressed genes; MEM, minimum essential medium; WT, wild-type; MUT, mutation-type; SD, standard deviation
