# Supplementary figures and images for: LINC01116 Promotes Doxorubicin Resistance in Osteosarcoma by Epigenetically Silencing miR-424-5p and Inducing Epithelial-Mesenchymal Transition
Source: Front Pharmacol. 2021 Mar 8;12:632206. doi: 10.3389/fphar.2021.632206 (PMC7982720; doi:10.3389/fphar.2021.632206)

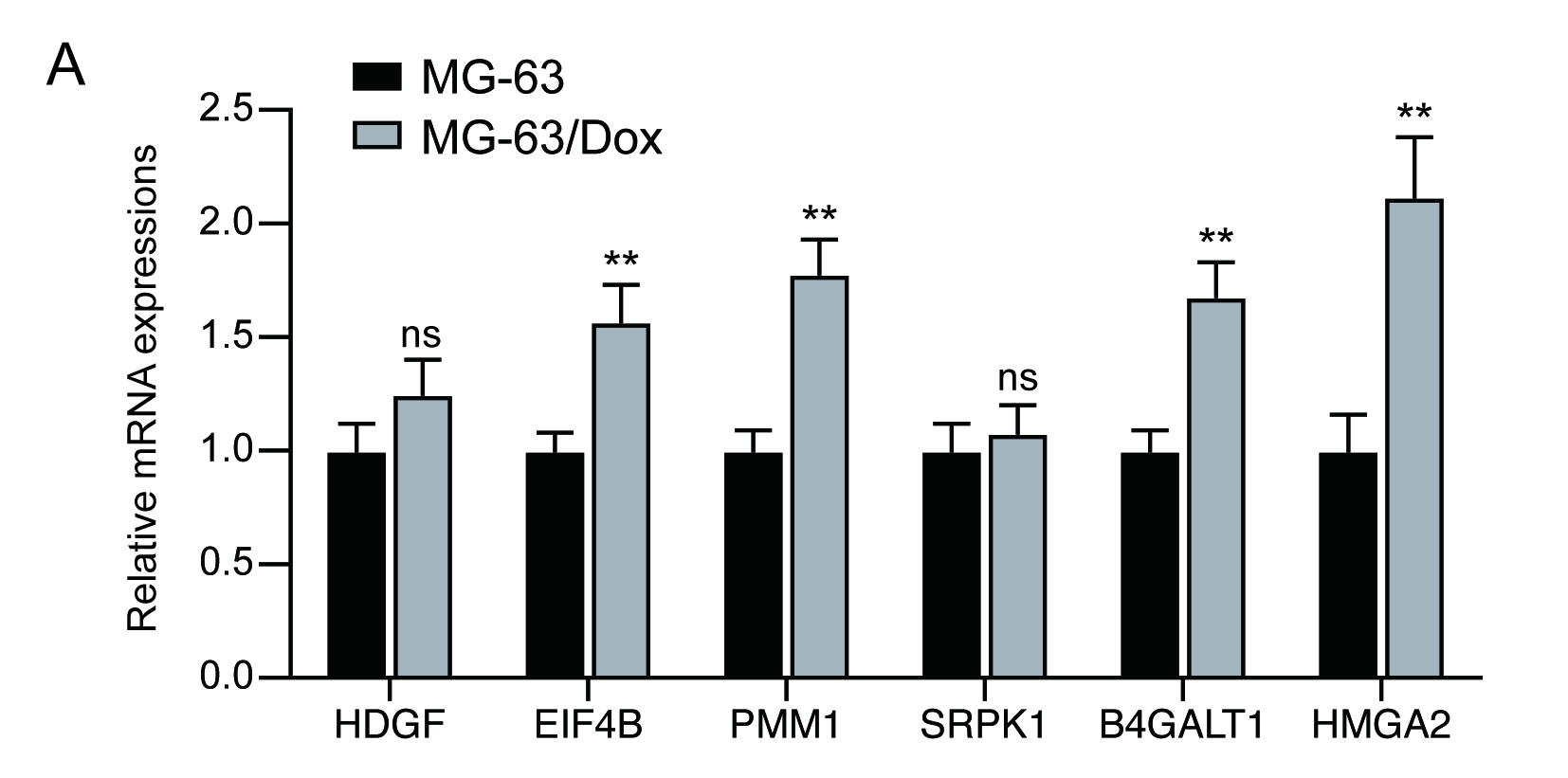

Supplement: Supplementary file 1 [file image1.tif]
