# Supplementary material for: LINC01116 Promotes Doxorubicin Resistance in Osteosarcoma by Epigenetically Silencing miR-424-5p and Inducing Epithelial-Mesenchymal Transition
Source: Front Pharmacol. 2021 Mar 8;12:632206. doi: 10.3389/fphar.2021.632206 (PMC7982720; doi:10.3389/fphar.2021.632206)
Supplement: Supplementary file 2 [file table1.docx]

**Table S1** shRNA, siRNA, mimics and inhibitor sequences

|  | Sequence |
| --- | --- |
| sh-LINC01116 | CACCGTGGTCCTTGACAGCCAATACGAATATTGGCTGTCAAGGACCA |
| sh-NC | CACCGTGGGTTCGACAACCCTATACGAATATAGGGTTGTCGAACCCA |
| si-EZH2 | GGATGGTACTTTCATTGAA |
| si-NC | GGAGTACTTTCATTTGGAA |
| miR-inhibitor | UUCAAAACAUGAAUUGCUGCUG |
| inhibitor-NC | UUCUCCGAACGUGUCACGUTT |
| miR-mimics | CAGCAGCAAUUCAUGUUUUGAA |
| mimics-NC | UUCUCCGAACGUGUCACGUTT |
