# Supplementary material for: LINC01116 Promotes Doxorubicin Resistance in Osteosarcoma by Epigenetically Silencing miR-424-5p and Inducing Epithelial-Mesenchymal Transition
Source: Front Pharmacol. 2021 Mar 8;12:632206. doi: 10.3389/fphar.2021.632206 (PMC7982720; doi:10.3389/fphar.2021.632206)
Supplement: Supplementary file 3 [file table2.docx]

**Table S2** Primers sequence for qRT‐PCR

|  | Forward primer | Reverse primer |
| --- | --- | --- |
| LINC01116 | CGCTTTGCTGAAGACGAGC | ATATTGAACTGAGCGGGGCT |
| HMGA2 | CTCAAAAGAAAGCAGAAGCCACTG | TGAGCAGGCTTCTTCTGAACAACT |
| HDGF | ATCAACAGCCAACAAATACC | TTCTTATCACCGTCACCCT |
| EIF4B | GGCAAAGCCTGTTGACACAGCT | TTCACTTCGCCAGCTTGGGTGT |
| PMM1 | GCATGAGCCACCACGCACAG | GGAGAGCCAGAGCCAGACAGAG |
| SRPK1 | GGTGTGCCAGTCTTCCTCAAC | GGTCCGTTATGTTCTTGCTCTTG |
| B4GALT1 | GGGCTCAGGTTCCAAGACTC | ACTACGTCAGCAAATGGGGG |
| miR-424-5p | CAGCAGCAATTCATGT | TGGTGTCGTGGAGTCG |
| GAPDH | GGAGCGAGATCCCTCCAAAAT | GGCTGTTGTCATACTTCTCATGG |
| U6 | CTCGCTTCGGCAGCACA | AACGCTTCACGAATTTGCGT |
