# Supplementary material for: LINC01116 Promotes Doxorubicin Resistance in Osteosarcoma by Epigenetically Silencing miR-424-5p and Inducing Epithelial-Mesenchymal Transition
Source: Front Pharmacol. 2021 Mar 8;12:632206. doi: 10.3389/fphar.2021.632206 (PMC7982720; doi:10.3389/fphar.2021.632206)
Supplement: Supplementary file 4 [file table3.docx]

**Table S3** Primers sequence for methylation-specific PCR

|  | Forward primer | Reverse primer |
| --- | --- | --- |
| miR-424-5p  (methylated) | GAGGCGTTGTTATATTTTTTCGT | GAACTTCCTTCTACTCCTAAACCG |
| miR-424-5p  (un-methylated) | GTGAGGTGTTGTTATATTTTTTTGT | CAAACTTCCTTCTACTCCTAAACCA |
